# Early high-sensitivity troponin elevation and short-term mortality in sepsis: a systematic review with meta-analysis

**DOI:** 10.1186/s13054-025-05249-2

**Published:** 2025-02-14

**Authors:** Abraham I. J. Gajardo, Santiago Ferrière-Steinert, Joaquín Valenzuela Jiménez, Sebastián Heskia Araya, Thomas Kouyoumdjian Carvajal, José Ramos-Rojas, Juan Nicolás Medel

**Affiliations:** 1 Intensive Care Unit, Department of Internal Medicine, Hospital Clínico Universidad de Chile, Dr. Carlos Lorca Tobar 999, 8380453 Independencia, Región Metropolitana, Santiago, Chile; 2 Program of Pathophysiology, Institute of Biomedical Science, Faculty of Medicine, Universidad de Chile, Santiago, Chile; 3 School of Medicine, Faculty of Medicine, Universidad de Chile, Santiago, Chile; 4 Dentistry School, Faculty of Medicine, Clínica Alemana-Universidad del Desarrollo, Santiago, Chile; 5 Epistemonikos Foundation, Santiago, Chile

**Keywords:** High-sensitivity troponin, Meta-analysis, Prognosis, Prognostic factor, Sepsis, Septic cardiomyopathy, Septic shock, Systematic review, Troponin

## Abstract

**Background:**

Serum cardiac troponin (cTn) elevation is a well-established phenomenon in sepsis. However, the clinical significance of this phenomenon with high-sensitivity (hs) assays and the current sepsis definition needs to be settled.

**Research Question:**

What is the association between early serum cTn levels measured by hs-assays and the risk of short-term mortality in septic patients?

**Study Design and Methods:**

We conducted a systematic review using a comprehensive PubMed, Scopus, and Embase search. Studies were eligible if they reported association data on early hs-cTn and mortality in an adult sample with sepsis that met the Sepsis-3 definition. For the synthesis of the effect of hs-cTn on mortality, we applied random effect models on the pooled unadjusted and adjusted odds ratio (OR and aOR, respectively) of elevated vs. normal hs-cTn serum values, and on the crude standardized mean difference (SMD) of hs-cTn between survivors and non-survivors.

**Results:**

In total, 6242 patients from 17 studies were included, with short-term mortality rates ranging from 16.9% to 53.8%. Using a crude analysis, non-survivor patients showed higher hs-cTn than survivors (SMD of 0.87, 95%CI: 0.41–1.33). Elevated hs-cTn was associated with increased mortality (OR = 1.78, 95% CI: 1.41–2.25). However, this prognostic effect was absent in studies that adjusted for different confounders (aOR = 1.06, 95% CI: 0.99–1.14).

**Discussion and Conclusions:**

Non-survivors of sepsis exhibited significantly elevated hs-cTn levels. While elevated hs-cTn levels are associated with an increased risk of mortality, they are not independently associated with this outcome in sepsis.

**Supplementary Information:**

The online version contains supplementary material available at 10.1186/s13054-025-05249-2.

## Background

According to the current definition (Sepsis-3), sepsis is a life-threatening organ dysfunction caused by a dysregulated host response to infection [1]. Patients diagnosed using the Sepsis-3 definition have a higher mortality risk and rate of adverse events [2, 3]; 25% of septic patients die in the hospital, increasing up to 50% in the septic-shock group [1]. Thus, sepsis is one of the most common causes of mortality in the intensive care unit (ICU) [3].

Sepsis can induce acute myocardial injury and cardiac dysfunction [4] and serum cardiac troponin (cTn) is used to diagnose acute myocardial injury [5]. In recent years, high-sensitivity cTn (hs-cTn) assays have increasingly replaced conventional essays because of their lower limit of detection and lower normality cutoff [6]. Currently, serum hs-cTn assessment is recommended to rule out acute myocardial infarction and to detect cardiac injury in other scenarios, such as sepsis [5, 7].

Although acute myocardial injury is well-established in patients with sepsis, the prognostic role of cTn elevation is unclear [4, 8, 9]. Previous meta-analyses [10–12] showed a significant association between cTn elevation and increased mortality in septic patients. However, these studies included patients who did not meet the current sepsis definition, were heterogeneous in timing and type of cTn assessment, were not oriented to a specific clinical setting, and did not control confounding factors [4]. Moreover, there are few studies on hs-cTn assays in the modern setting, which may impact the stratification of patients and, therefore, the prognostic association of this biomarker with mortality risk [7]. Thus, we conducted this systematic review to assess the association of early serum hs-cTn levels with short-term mortality of patients admitted to the ICU or the emergency department (ED) because of sepsis, according to Sepsis-3.

## Methods

The methodology was defined a priori, and it is fully described in a published protocol [13], which was created using the standard guidelines for systematic reviews of prognostic factors [14] and is registered in PROSPERO (CRD42024468883) in alignment with the Preferred Reporting Items for Systematic review and Meta-Analysis (PRISMA) statement [15]**.**

### Eligibility criteria

Both observational and interventional primary studies were deemed eligible if they included at least ten events in adult patients diagnosed either with *sepsis* by Sepsis-3 criteria [1], *severe sepsis* by Sepsis-1 or Sepsis-2 [16, 17], or *septic shock* by any of the above criteria [1, 16, 17]. Additionally, studies were not eligible if they focused exclusively on patients with a specific underlying pathology, such as cancer. Eligibility was further restricted to studies that specified the utilization of any hs-cTn assay within the first 24 h and whose reported limit of detection was considered high-sensitivity (i.e., < 5 ng/L) [5]. When this limit was not provided, we excluded studies that failed to meet a normal limit cutoff of 20 ng/L or lower [5]. Only studies conducted in ICU or ED settings that evaluated short-term mortality (defined as reported ICU, 28-day, or in-hospital mortality; in that preferential order) were included.

### Information sources and search strategy

In August 2024, PubMed, Scopus, and Embase were searched for primary articles with no date limit and no language restriction. The search strings combine free text and MeSH/EMTREE terms for all words related to troponin and sepsis [13]. Also, we manually scrutinized references of previous reviews to retrieve missing pertinent papers. Abstracts and conferences were suitable for inclusion only if they provided all critical data. Gray literature and retracted papers were not included.

### Selection process and data collection

Two independent reviewers scrutinized the search results, and a third resolved any discrepancies. Studies that passed a first screening based on the title and abstract under criteria were extracted for full-text assessment. The data collection process was conducted using standardized forms. Reported associations suitable to be extracted were: (1) dose–response: odds ratios (OR), risk ratios (RR), or hazard ratios (HR) per unit increase in cTn; (2) category-or quantile-based data: contingency tables or ratios by cTn level groups; (3) means: means or mean differences in cTn between survivor and non-survivor groups [18]. Adjusted and unadjusted effect measures were collected separately. Authors of each study were contacted to request critical missing data regarding these associations. OR was used as a common effect measure for dose–response and category/quantile-based data, as we previously described [13].

### Quality assessment

Two reviewers independently assessed the risk of bias (RoB) by applying the Quality in Prognostic factor Studies (QUIPS) tool [19], which considers the following domains: (1) study participation, (2) study attrition, (3) prognostic factor measurement, (4) outcome measurement, (5) study confounding, and (6) statistical analysis and reporting. The overall assessment could be categorized as low, moderate, or high RoB. A third reviewer resolved discrepancies. We predefined D1, D3, and D5 as the key domains for our assessment and specified criteria for each of these domains (Additional File 1). Predetermined core adjustment factors in the confounding domain were: severity of sepsis, age, and clinical comorbidity (any cardiac or renal). Certainty of evidence was assessed with GRADE guidelines for prognostic studies [20].

### Statistical analysis

The structural analysis of mortality risk considered separate models for unadjusted and adjusted effect measures. Regarding the unadjusted model, whenever feasible, we built a 2 × 2 contingency table [7, 21–27] from data, according to the cutoff defined by each study; otherwise, we directly integrated extracted ORs with their standard errors (SEs) [28] into the model. The adjusted model incorporated effect measures from category-based (dichotomous variable) [7, 22, 27, 28] and dose–response (continuous variable) multivariate regressions [24, 29, 30]. As predetermined [13]**,** dose–response ORs were scaled to an OR per 50 ng/L increase [29, 30], from a 10 ng/L baseline in the case of log-transformed cTn variable [24], since we considered this magnitude useful and representative of the difference between normal and elevated cTn groups [24, 25, 27]. We handled one extracted HR [27] as OR because its its value was close to 1 [31].

We performed the meta-analysis of studies reporting serum troponin mean and standard deviation (SD) data by survival status with Hedges´g standardized mean difference (SDM) [32]. Means and SDs were estimated from quartile data [33] when not explicitly reported [7, 21, 25–27, 34–36].

All pooled estimates were computed by inverse variance weighting in random effect models using the R-4.3.1 package *metafor* [37]. SEs were obtained from *p*-values [38]. Raw data, calculations, and formulas can be accessed in Additional File 1. We adopted a minimally contextualized approach to interpreting the prognostic value of hs-cTn using a clinically relevant threshold for elevated hs-cTn levels. In line with this, the threshold for clinical relevance was set using the relative estimator, with an OR value of 1 and a SMD of 0 as the threshold for no effect.

## Results

### Selected studies

**Fig. 1:**
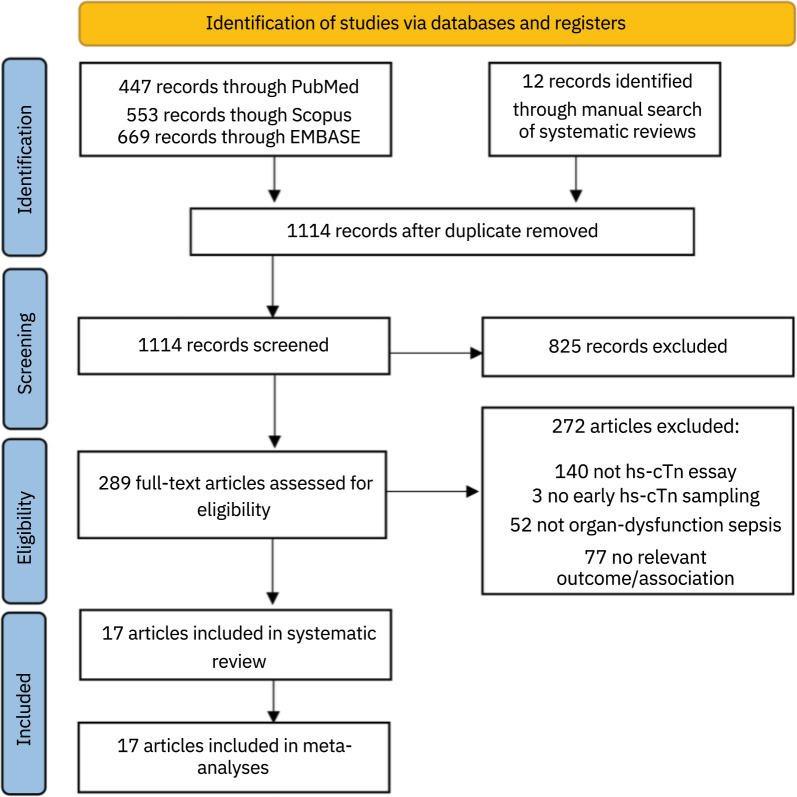
Flowchart of study selection process. hs-cTn: High-sensitivity cardiac troponin

After discarding duplicates, we obtained 1114 references, of which 289 passed the primary screening, and 17 were finally included [7, 21–30, 34–36, 39–41], encompassing 6242 patients (Fig. 1). Patients’ mean age ranged between 57 and 73.3. The male percentage was between 52 and 70%. Regarding the type of hs-cTn, 12 studies used cTnT [7, 24, 25, 27, 28, 30, 34–36, 39–41], and five used cTnI [21–23, 26, 29]; none included both.

Mortality follow-up was 28 to 30 days in 10 studies [21–23, 26–29, 34, 39, 40], up to ICU discharge in two studies [25, 30], and in-hospital in five studies [7, 24, 35, 36, 41]. The short-mortality rate spanned between 16,9% and 53,8%. The sampling time of troponin was described as during the first day in three studies [7, 35, 41], and at-admission in the rest. Three studies were multi-center and 14 single-center. Concerning the study type, 11 studies were prospective cohorts [7, 21, 22, 26, 28, 34–36, 39–41], four historical cohorts [24, 25, 27, 29], and two randomized clinical trials [23, 30] (Table 1).

**Table 1:** Included studies

Author, year	Patients (n)	Setting, Country	Excluded comorbidities	Study design	Sepsis criteria, % Shock	Mortality Rate, Follow-up Mortality	Troponin type and cut-off	Variables in multivariate analysis	Reported associations
Innocenti et al. (2022) [21]	164	ED, Italy	Cardiovascular	Prosp.	Sepsis and septic shock (Sepsis-3), 41% shock	29.5%, 28-day	cTnI (100 ng/L)	NA	Contingency table, Mean difference (a)
Landesberg et al. (2012) [35]	262	ICU, NR	Cardiovascular	Prosp.	Severe sepsis and septic shock (Sepsis-1), 62% shock	36%, In-hospital	cTnT (30 ng/L)	NA	Mean difference
Røsjø et al. (2011) [7]	204	ICU, Finland	None	Prosp.	Severe sepsis and septic shock (Sepsis-1), % shock NR	22,70%, In-hospital	cTnT (14 ng/L)	cTnT, Age, CVD, eGFR, Lactate, SAPS II, SOFA	Contingency table, Mean difference, Multivariate regression
Vallabhajosyula et al. (2017) [24]	944	ICU, USA	None	Retro	Severe sepsis and septic shock (sepsis-2), 66% shock	25,90%, In-hospital	cTnT (10 ng/L)	log10(cTnT), age, sex, BMI, Charlson index, AKI, Resp. failure	Contingency table, Multivariate regression
Landesberg et al. (2015) [36]	105	ICU, NR	Cardiovascular	Prosp.	Severe sepsis and septic shock (sepsis-1), 71% shock	42%, In-hospital	cTnT (NA)	NA	Mean difference
Masson et al. (2016) [30]	955	UCI, Italy	Traumatic, Cardiovascular, Hepatic, Renal	RCT	Severe sepsis and septic shock (sepsis-1), 56% shock	26,40%, ICU	cTnT (26 ng/L)	cTnT, Age, sex, BMI, reason for admission to ICU, MV, SAPS II, SOFA, COPD, CKD, immunodeficiency, HF, Pao2/FIO2, platelet count, bilirubin, lactate, diuresis	Multivariate logistic regression
Antcliffe et al. (2019) [23]	442	ICU, UK	Renal, Hepatic, Cardiovascular	RCT	Septic shock (sepsis-2), 100% shock	27,10%, 28-day	cTnI (34 ng/l)	NA	Contingency table
Sasko et al. (2015) [22]	50	ICU, Germany	None	Prosp.	septic shock (Sepsis-2), 100% shock	53,80%, 28-day	cTnI (15 ng/L)	cTnT > 15 (ng/L), sex, age, CAD, MAP < 65, CVP < 12, SvO2 < 70%, HTC < 30%, LVEF < 40%	Contingency table, Mean difference, multivariate logistic regression
De Geer et al. (2015) [34]	50	ICU, Sweden	None	Prosp.	septic shock (Sepsis-2), 100% shock	30%, 30-day	cTnT (NA)	NA	Mean difference
Sirvent et al. (2015) [39]	42	ICU, Spain	None	Prosp.	sepsis and septic shock (sepsis-2), 69% shock	35,70%, 28-day	cTnT (NA)	NA	Mean difference
Kim et al. (2020) [29]	778	ED, South Korea	Cardiovascular	Retro	Septic shock (Sepsis-2), 100% shock	28,70%, 28-day	cTnI (40 ng/L)	cTnI, hypertension, DM, CKD, malignancy, gastrointestinal infection, unknown site infection, RV dysfunction	Multivariate logistic regression
Lundberg et al. (2016) [40]	53	ICU, Sweden	Hematological, Immunological	Prosp.	Septic shock (Sepsis-2), 100% shock	28,30%. 28-day	cTnT (14 ng/L)	NA	Mean difference
Landesberg et al. (2014) [41]	106	ICU, Israel	Cardiovascular	Prosp.	Severe sepsis and septic shock (sepsis-2), 61% shock	38.6%, In-hospital	cTnT (14 ng/L)	cTnT, RVESV, longitudinal SRe’	Univariate and Multivariate logistic regression, Mean difference
Wang et al. (2020) [28]	98	ICU, China	Cardiovascular, Immunosuppression	Prosp.	Sepsis and septic shock (Sepsis-3), 43% shock	26.5%, 28-day	cTnT (100 ng/L)	cTnT > 100 (ng/L), hs-CRP, APACHE-II, SVI, APN, Cardiac index	Univariate and Multivariate logistic regression
Jendoubi et al. (2019) [26]	75	ICU, NR	Cardiovascular	Prosp.	Septic shock (Sepsis-1), 100% shock	54.6%, 28-day	cTnI (34 ng/L for men and 16 ng/L for women)	NA	Contingency table, Mean difference
Vallabhajosyula et al. (2018) [25]	602	ICU, US	Renal, Cardiovascular	Retro	Severe sepsis and septic shock (Sepsis-2), 69% shock	17.4%, ICU	cTnT (10 ng/L)	NA	Contingency table, Mean difference (b)
Xu et al. (2023) [27]	1312	ICU, China	Cardiovascular	Retro	Sepsis and septic shock (sepsis-3), 59% shock (c)	29,30%, 28-day	cTnT (10 ng/L)	cTnT > 10 (ng/L), Hypertension, DM, CKD, SOFA, mechanical ventilation, CRRT, Vasoactive support, SIMI, creatinine	Contingency table, univariate Cox regression, multivariate Cox regression

a Provided by correspondence; b Extracted from Individual patient data in the supplement; c Extrapolated from vasoactive support prevalence. NA: Not Applicable. NR: Not Reported. Retro: Retrospective. Prosp: Prospective. RCT: Randomized Controlled Trial. cTnT: Cardiac Troponin T. CVD: Cardiovascular Disease. eGFR: Estimated Glomerular Filtration Rate. SAPS II: Simplified Acute Physiology Score II. SOFA: Sequential Organ Failure Assessment. BMI: Body Mass Index. APACHE-III: Acute Physiology and Chronic Health Evaluation III. AKI: Acute Kidney Injury. MV: Mechanical Ventilation. COPD: Chronic Obstructive Pulmonary Disease. CKD: Chronic Kidney Disease. HF: Heart Failure. Pao2/FIO2: Partial Pressure of Arterial Oxygen/Fraction of Inspired Oxygen. MAP: Mean Arterial Pressure. CVP: Central Venous Pressure. SvO2: Mixed Venous Oxygen Saturation. HTC: Hematocrit. LVEF: Left Ventricular Ejection Fraction. cTnI: Cardiac Troponin I. DM: Diabetes Mellitus. RV: Right Ventricle. RVESV: Right Ventricular End-Systolic Volume. SRe’: Longitudinal Systolic Strain Rate. hsCRP: High-Sensitivity C-Reactive Protein. APACHE-II: Acute Physiology and Chronic Health Evaluation II. SVI: Stroke Volume Index. APN: Acute Pulmonary Nodule. CRRT: Continuous Renal Replacement Therapy. SIMI: Sepsis-Induced Myocardial Injury

### Risk of bias

In the adjusted mortality risk assessment, overall RoB was low in all but two studies included in the model [28, 29]. The study confounding domain was the most problematic since these studies did not adjust for core factors [28, 29]. Other risks were deemed minor and did not justify of downgrading the overall judgment of the study as predefined in the protocol [14]. The RoB assessment of unadjusted OR and SMD models can be accessed in the Additional File 1.

### Meta-analysis

**Fig. 2:**
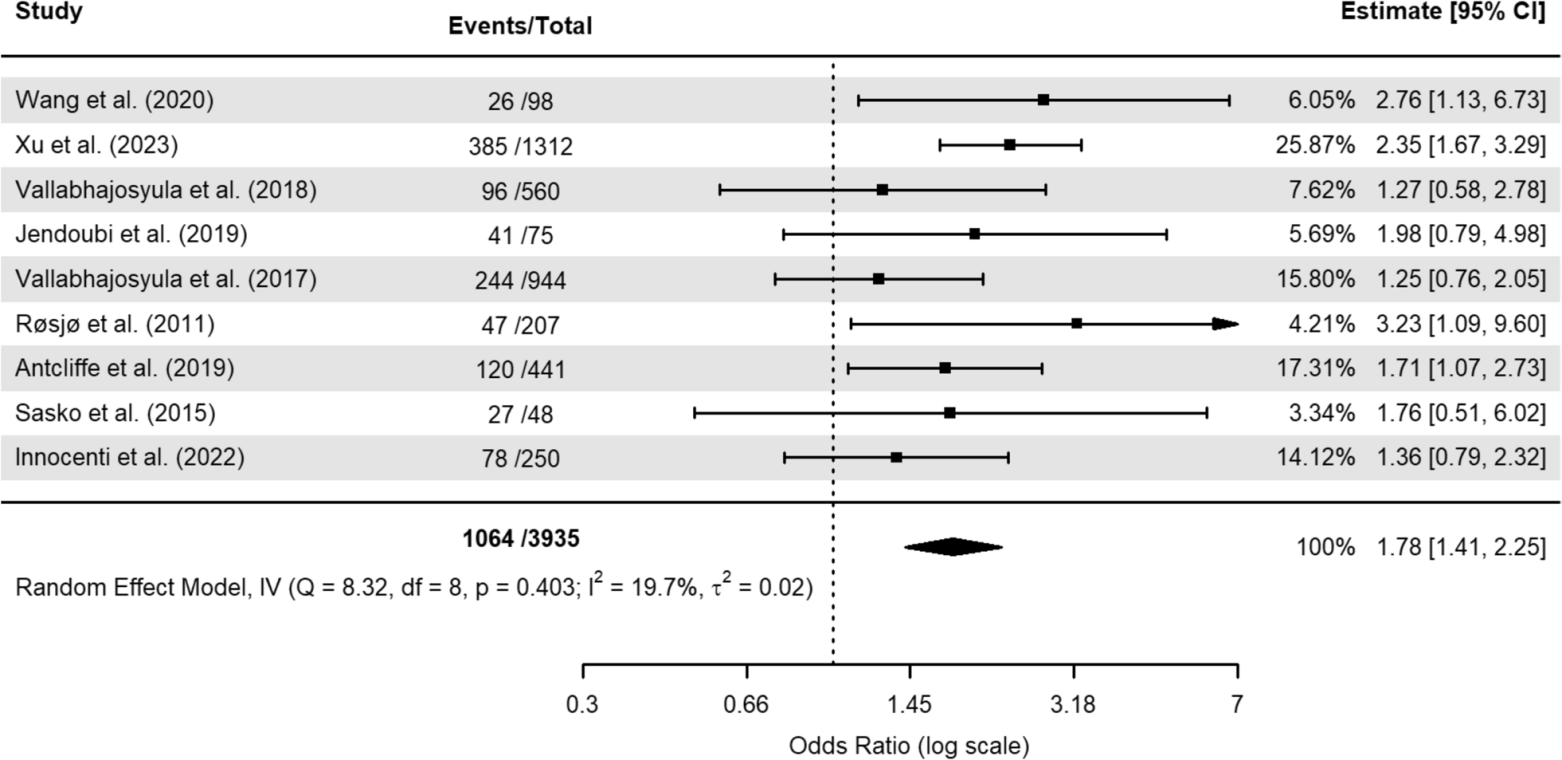
Meta-analysis of unadjusted association between elevated high-sentivity cardiac troponin and sepsis mortality

**Fig. 3:**
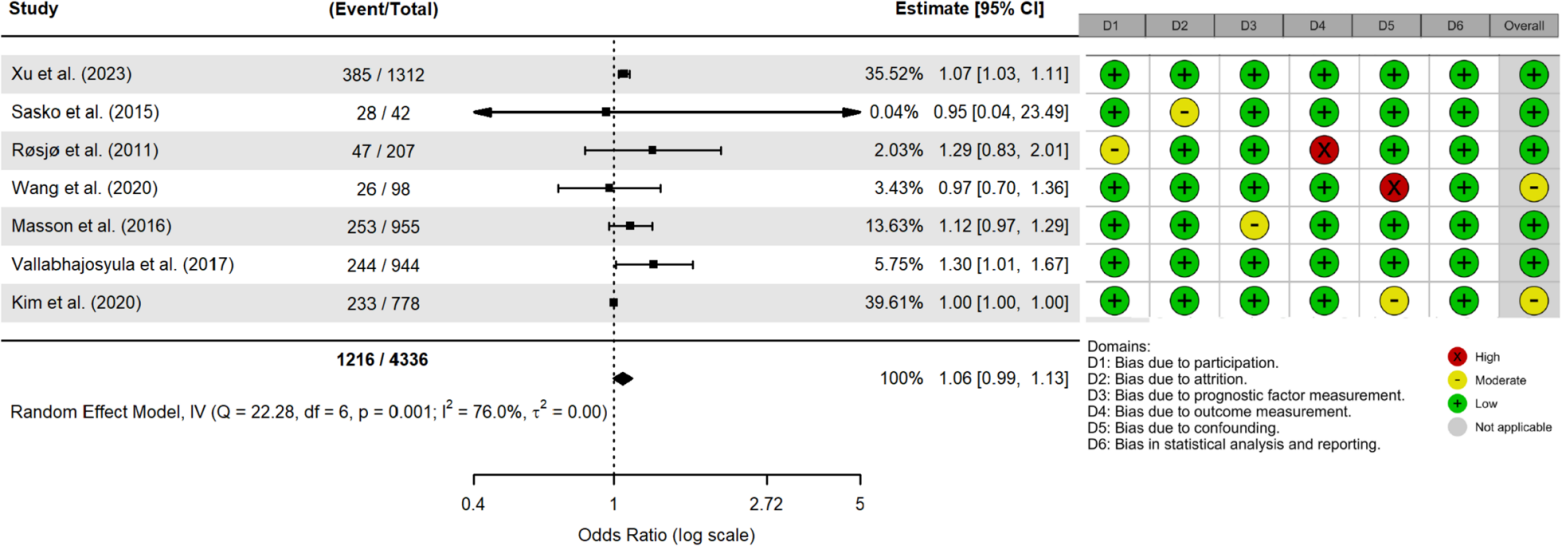
Meta-analysis of the adjusted association between elevated high-sentivity cardiac troponin and sepsis mortality

In the mortality odds unadjusted model, a total of 1064 individuals died amongst 3935 patients, constituting a mortality rate of 27%. The pooled OR for elevated hs-cTn was 1.78 (95% confidence interval (CI): 1.41–2.25), which suggests that individuals in the elevated troponin group had 78% higher odds of death (Fig. 2). The adjusted model included 1216 events out of 4336 total patients, with a mortality rate of 28%. The adjusted model showed no significant association between elevated hs-cTn and mortality (OR = 1.06, 95% CI 0.99–1.13). We omitted one study [41] from the latter model because it reported an OR that was thousands of orders of magnitude greater than the others (Fig. 3.). Disease severity scores (SOFA, APACHE-II, and SAPS-II), comorbidities, age, and cardiac function parameters, among others, were common confounding factors used to adjust the effect estimates (Table 1). The certainty of the evidence was assessed using the adjusted analysis and was deemed to be moderate (Table 2).

**Table 2:** Summary of Findings

No of studies (Events/Participants)	Certainty assessment						Effect			Certainty
	Study design	Risk of bias	Inconsistency	Indirectness	Imprecision	Publication bias	Mortality rate without prognosis factor	Mortality rate with prognosis factor	Rate (95% CI)	
Adjusted Odd Ratio for Short-Term Mortality in Organ-Dysfunction Sepsis Patients with Elevated Early High-Sensitivity Troponin										
7 (1216/4336)	non-randomized studies	not serious	not serious^a^	not serious	serious^b^	none	17.1%^c^	17.9%	Adjusted Odds Ratio 1.06 (0.99 to 1.13)	⨁⨁⨁◯ Moderate

Question: Association of Short-Term Mortality and Early High-Sensitivity Troponin in Organ-dysfunction Sepsis

Setting: Organ-dysfunction sepsis

^a^Despite high statistical heterogeneity, we did not downgrade for heterogeneity as it was attributed to the large sample size, and the sensitivity analysis showed consistent results

^b^Since the confidence interval of the pooled estimate does not exclude the null effect, it was considered a downgrading factor

^c^Data from Xu et al. (2023) study

**Fig. 4:**
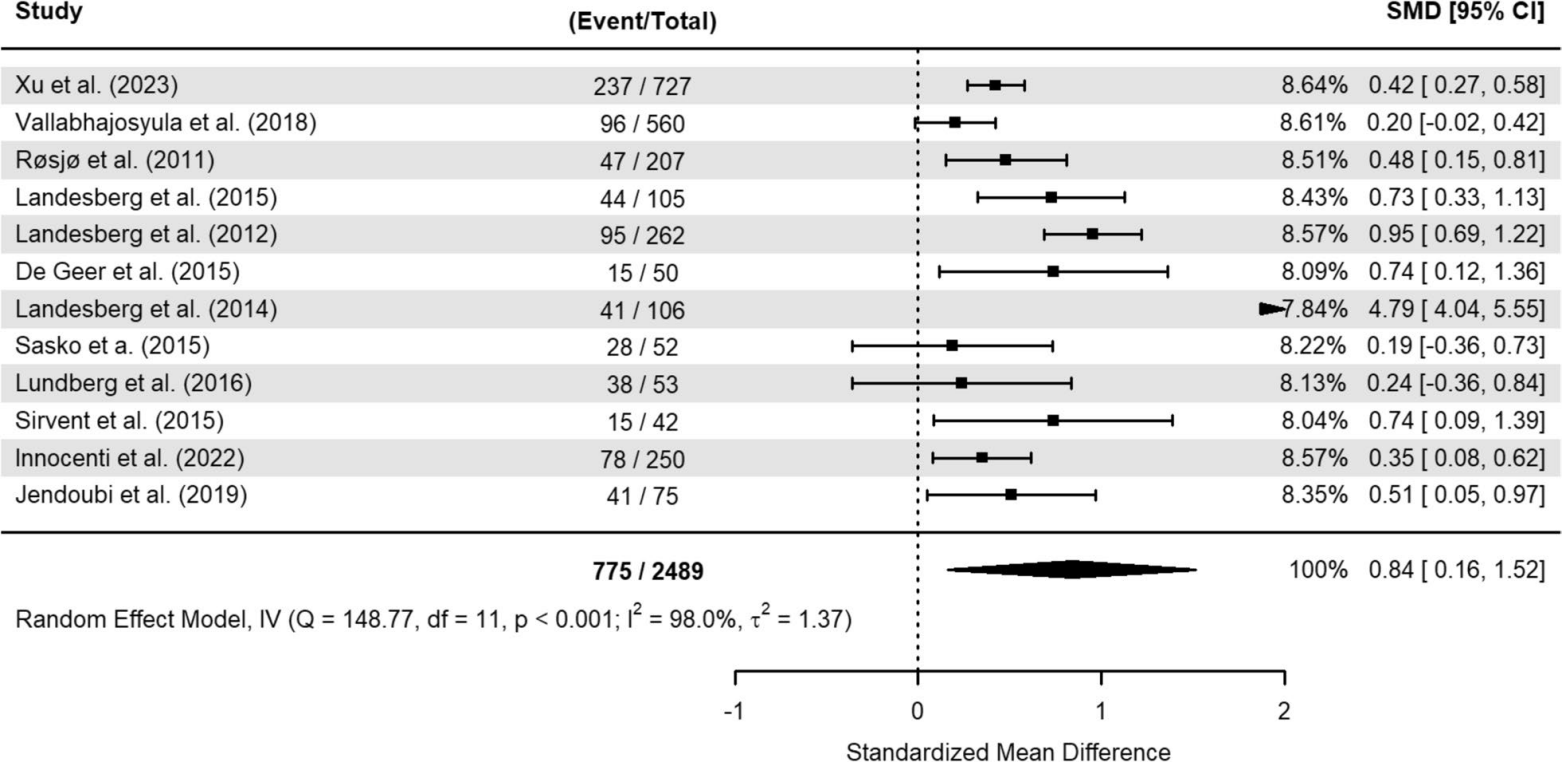
Meta-analysis of standardized mean difference of serum high-sensitivity cardiac troponin between survivors and non-survivors

In the SMD model, the incidence of events was 31% (775 out of 2489 patients). This unadjusted estimate revealed an increase of 0.84 SMD (95% CI 0.16–1.52) of serum hs-cTn levels among the deceased patients (Fig. 4.). Using a representative standard deviation [27], this effect size would translate to a mean difference of 105.6 ng/L (95% CI: 20.4 to 190.8 ng/L) between both groups.

### Heterogeneity and sensitivity analysis

We assessed inconsistency primarily by visual inspection and then taking into consideration I [2] statistics (where I2 > 50% and *p*-value < 0.10 is usually considered *relevant heterogeneity*) [20]. The unadjusted mortality odds model barely showed any heterogeneity at all. In the adjusted odds model, we attributed the high I2 statistic to the large sample size and small CIs of the two main studies [27, 29]. Indeed, by removing any of the studies, the I2 statistic dropped to 44.5% and 0%, respectively. Ultimately, we deemed heterogeneity irrelevant since their estimates were very close.

A sensitivity analysis of only with category-based adjusted effect measures [7, 22, 27, 28], presented an estimate reasonably similar to the model with all adjusted studies (OR 1.07, 95% CI: 1.03–1.13). Notably, this estimate also almost exclusively renders a low RoB sample. Therefore, no subgroup analyses were performed on these models.

The troponin SMD model revealed substantial heterogeneity by visual and statistical tests. Subgroup analysis for cohorts of patients with shock vs. without shock, and by troponin assay could not identify the root of the heterogeneity (*p*-values for subgroup differences were 0.87 and 0.73, respectively). When we removed the Landesberg et al. study [41] because its SMD and adjusted OR were far greater than the rest of the studies, the estimate decreased to 0.49, but I2 only reduced to 60%. The forest plots of subgroup analyses are accessible in the Additional File 1.

### Publication bias

**Fig. 5:**
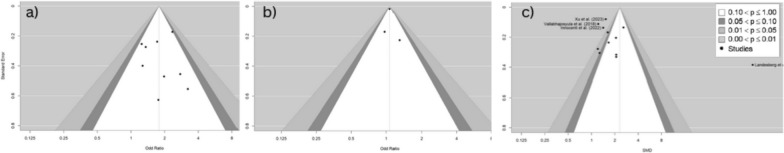
Funnel plots for **a** unadjusted mortality risk model; **b** adjusted mortality risk model; **c** troponin SMD model

Despite having few studies in each model, none of the funnels presented asymmetry. Considering the broad search, which included abstracts and conferences, we have no reason to think that any estimates have been systematically omitted (Fig. 5). We ran an Egger test for the SMD model, which has over ten studies, and was not statistically significant.

## Discussion

This systematic review with meta-analysis assessed the association between hs-cTn and short-term mortality in ICU/ED patients with sepsis according to the current definition. Although non-surviving patients showed a significant increase in early hs-cTn levels, this increase probably resulted in little to no difference in mortality after being controlled for confounding factors.

We anticipated that myocardial injury, assessed by hs-cTn, could improve prognostic value in septic patients. Acute myocardial injury in sepsis is multifactorial, involving direct inflammatory damage to cardiomyocytes and myocardial oxygen demand/supply mismatch (type 2 myocardial infarction), and can even lead to type 1 myocardial infarction (atherosclerotic plaque disruption) [42]. Altered kidney function can also decrease the cTn clearance after a cardiac insult in ICU patients [43]. In this context, because hs-cTn has a lower detection limit and lower normality cutoff than conventional cTn assays [6], we hypothesized that hs-cTn assays would allow earlier and better detection of myocardial injury in sepsis, which in turn would increase its relationship with mortality. In fact, a better prognostic value of hs-cTn compared with conventional cTn assays has been well-established in patients with acute myocardial infarction [44, 45], suggesting similar findings in other myocardial injury etiologies, such as sepsis.

Our results differ from previous meta-analyses, which have consistently shown a significant association between cTn and sepsis mortality [10–12]. However, two of these studies were published before Sepsis-3 and included a few studies using hs-cTn [10, 11]; all three did not exclusively include patients with organ dysfunction, which is mandatory in the current sepsis definition. Only one previous meta-analysis reported an effect estimate adjusted for confounders [11]. Furthermore, all these systematic reviews included studies that withdrew blood samples for cTn mostly early [12] or were exclusively early (hospital admission) [10, 11]. Thus, the present systematic review with meta-analysis represents an updated evidence synthesis that aligns with the current definitions of sepsis and recommended clinical practice [1, 5].

The stricter criteria of Sepsis-3 restricts sepsis diagnosis to patients with acute organ dysfunction [1], making the sepsis entity closer to severe sepsis in earlier criteria. Given that the Sepsis-3 diagnosis correlates with a higher risk of adverse events than earlier consensus diagnoses [2], comparing the predictive value of cTn from previous systematic reviews undertaken with older sepsis definitions is difficult. Moreover, the sensitivity of the cTn essay could significantly affect the biomarker's prognostic ability. For instance, Røsjø et al. reported a significant unadjusted association for hs-cTnT but not for 4th-gen cTnT and mortality [7]. Thus, the differences between the results of our systematic review and those of previous ones may be explained by the inclusion of more severely ill patients and higher sensitivity of cTn essays in our study than in others.

The effect of confounding factors on the association of interest was also considered in our study. Our meta-analysis of unadjusted mortality measures yielded an OR of 1.78 (95% CI: 1.41–2.25), which is comparable with the estimates reported in the literature (RR = 1.91 and HR = 1.35, respectively) [10–12]. Nevertheless, our adjusted model included some 4336 patients and showed an OR of 1.06 (95% CI: 0.99–1.13), indicating that cTn loses much of its prognostic value once the data are controlled for well-known and usually assessed clinical factors. This finding sharply contrasts with the review by Sheyin et al. [10], in which the estimate remained significant in the multivariate group sensitivity analysis. Bessière et al. reported a similar finding, presenting an adjusted OR of 1.92 (95% CI: 1.35–2.74) for 791 patients [11]. However, none of the four studies included in this model fell under our PICOTS, and the most recent was published in 2010. Many of the adjustment factors are similar in our selected studies (age, severity scores, comorbidities, and biomarkers), which might imply that restricting studies to a setting of high-sensitivity assays and/or organ-dysfunction sepsis affects the prognostic ability of the cTn biomarker.

The evidence presented here helps physicians better interpret hs-cTn elevation in septic patients from a clinical perspective. Considering that cTn elevation is common in septic-shock patients [24], and that hs-cTn plasma levels were higher in non-survivors than surviving patients, this confirms that myocardial injury is an early and frequent condition in septic patients that later suffer clinical deterioration. Consequently, a high hs-cTn plasma value in a septic patient should alert clinicians of an increased risk of adverse outcomes. Although the association between high hs-cTn and mortality was no longer significant in the meta-analysis of adjusted estimates (wherein all included studies controlled for organ dysfunction scores), this could reflect that an elevated hs-cTn in sepsis is also related to global organ dysfunction and not only to myocardial injury alone. Thus, even without an independent association with mortality, hs-cTn levels are a valuable biomarker for identifying patients at higher risk of adverse outcomes and could play an important role in clinical risk stratification. Indeed, how hs-cTn temporal trajectories and peak values could affect sepsis prognosis and whether hs-cTn improvement translates into better outcomes for patients with sepsis need further investigation. Finally, although this study confirms the prognostic utility of a widely available biomarker, the prognostic significance of hs-cTn compared with other promising biomarkers in sepsis, such as suPAR [46] and proADM [47], could be the subject of further studies.

### Limitations

While we found no evidence of publication bias in any of the models, the lack of specification of the hs-cTn assay limit of detection in many studies was a limitation for study selection in our review; in fact, many studies could have been discarded based on this issue. Also, the disparity in hs-cTn normality cutoff among primary studies might drastically impact the effect measures after dichotomization (elevated vs normal groups). Finally, a main limitation in the meta-analysis of adjusted models is the discrepancy in the confounder factors set and the omission of relevant variables in some regressions from primary studies.

We also recognize that using aggregate data limits the ability to fully control for confounding despite including available adjusted estimates. While individual patient data (IPD) meta-analysis might offer better confounding control, it faces significant practical challenges and cannot eliminate residual confounding or address other types of bias inherent to observational studies [48]. Thus, our systematic review provides reliable evidence within the methodological constraints of evidence-based medicine.

On the other hand, chronic cardiovascular conditions may affect baseline high-sensitivity troponin levels in septic patients. However, most studies in this systematic review excluded patients with pre-existing cardiovascular disease to minimize confounding. Future research should address how pre-existing cardiovascular conditions modify the prognostic value of high-sensitivity troponin in sepsis.

## Conclusions

This systematic review with meta-analysis provides a valuable, generalizable, and updated insight into the clinical practice of sepsis. Our study shows that elevated hs-cTn levels in septic patients are associated with an increased risk of short-term mortality, highlighting their utility as a widely available biomarker for risk classification in sepsis. Nevertheless, after controlling for confounding factors, the association between early hs-cTn elevation and short-term mortality is attenuated, suggesting that hs-cTn is not independently associated with mortality risk in sepsis under the current definition.

## Supplementary Information

Additional file 1. 

## Data Availability

All data generated or analysed during this study are included in this published article and its supplementary information files.

## Abbreviations

aOR: Adjusted odds ratio
ED: Emergency department
HR: Hazard ratio
hs-cTn: High-sensitivity cardiac troponin
ICU: Intensive care unit
RoB: Risk of bias
RR: Risk ratio
SMD: Standardized mean difference
uOR: Unadjusted odds ratio

## Declarations

**Ethics approval and consent to participate**

Not applicable.
