# Supplementary material for: Early high-sensitivity troponin elevation and short-term mortality in sepsis: a systematic review with meta-analysis
Source: Crit Care. 2025 Feb 14;29:76. doi: 10.1186/s13054-025-05249-2 (PMC11829436; doi:10.1186/s13054-025-05249-2)
Supplement: Supplementary file 1 — Additional file 1. [file 13054_2025_5249_MOESM1_ESM.pdf]

Online Appendix

Abraham I. J. Gajardo, Santiago Ferrière-Steinert, Joaquín Valenzuela Jiménez, Sebastián Heskia Araya, Thomas Kouyoumdjian Carvajal, José Ramos-Rojas, Juan Nicolás Medel.

- Introduction
- Search Strategies
- Data Synthesis
- Unadjusted meta-analysis
  - Load arm-based data extraction
  - Load contrast-based data extraction
  - Meta-analysis model of pooled ORs
    - Studies data
- Adjusted meta-analysis
  - Load data and Homogenization of dose-response effect measures
  - Model creation
  - Sensitivity analysis
    - Studies data
- Mean difference data meta-analysis
  - Load data
  - Calculation of Arithmetic statistics
  - SMD model
  - Interpret the model
  - Heterogeneity investigation
    - Studies data
- Publication Bias
- Risk of Bias assesment algorithm
- References

Introduction

The dataset for each code segment is provided at the end of the corresponding section. Before executing the code, it is necessary to install the following R packages:Metafor, Robvis, Readxl, Dplyr, Knitr, and Ggplot.

Search Strategies

Pubmed string ((((“troponin i”[MeSH Terms]) OR (“troponin”[MeSH Terms])) OR (“troponin c”[MeSH Terms])) OR(“troponin t”[MeSH Terms]) OR (“troponin*“[All Fields]) AND ((humans[Filter]))) AND (((”sepsis”[MeSH Terms]) OR (“shock, septic”[MeSH Terms])) OR (Systemic Inflammatory Response Syndrome[MeSH Terms]) AND ((humans[Filter])))

Scopus string (TITLE-ABS-KEY (troponin) AND TITLE ((sepsis) OR (septic AND shock)) AND KEY ((sepsis) OR(septic AND shock)))

EMBASe string ’sepsis’/exp/mj AND troponin* AND [humans]/lim

Data Synthesis

Unadjusted meta-analysis

For the meta-analysis of unadjusted odds ratios (ORs), we will load data from the following sources: 1) Studies providing 2x2 contingency tables, which are considered the gold standard for unadjusted ORs in this review (arm-based data extraction). 2) Effect measures reported directly within study reports (contrast-based data extraction).

Load arm-based data extraction

```
#Load 2x2 contingency tables
data <- read_excel("contingency.xlsx")

#Derive the OR from the table
es_contingency <- escalc(measure = "OR", ai = high_tn_diseased, bi = high_tn_survived, ci = low_tn_diseased , di
= low_tn_survived, data = data)

#Include sample size in the data frame
es_contingency <- es_contingency %>%
  group_by(study) %>%
  mutate(n = sum(high_tn_diseased, high_tn_survived, low_tn_diseased, low_tn_survived))
```

Load contrast-based data extraction

This step involves extracting the effect measure directly along with its corresponding p-value. Given that meta-analysis models utilize the natural logarithm of ratio effect measures, the standard error of the log(OR) was calculated as follows **(1)**:

$$SE(\hat{\beta}_1) = \frac{|\hat{\beta}_1|}{Z_{(1-\frac{p}{2})}}$$

```
#load the data
data_contrast <- read_excel("contrast.xlsx")

# Assuming that the bounds of confidence interval were obtained from a Wald-type CI, we can use the following met
afor function:
es_contrast <- conv.wald(out= OR, pval = p, n = n, data=data_contrast, transf=log)
```

Meta-analysis model of pooled ORs

With the ORs obtained for all unadjusted studies, a random-effects model (rma) will be employed for the meta-analysis, utilizing inverse variance weighting.

```
#Combine all studies
es_combined <- bind_rows(es_contingency, es_contrast)

#Define new variables for the plot
es_combined <- es_combined %>%
  mutate(events = coalesce(low_tn_diseased, 0) + coalesce(high_tn_diseased, 0) + coalesce(diseased_n, 0))
total_n <- sum(es_combined$n)
total_events <- sum(es_combined$events)

#Random Effect Model
model_combined <- rma(yi, vi, data = es_combined, method = "REML", slab = es_combined$study)

#Describe function to add heterogeneity test
mlabfun <- function(text, x) {
  list(bquote(paste(.(text),
                    " (Q = ", .(fmtx(x$QE, digits=2)),
                    ", df = ", .(x$k - x$p), ", ",
                    .(fmtp(x$QEp, digits=3, pname="p", add0=TRUE, sep=TRUE, equal=TRUE)), "; ",
                    I^2, " = ", .(fmtx(x$I2, digits=1)), "%, ",
                    tau^2, " = ", .(fmtx(x$tau2, digits=2)), ")")))}

#Plot
forest(model_combined, slab = es_combined$study, atransf = exp,
       showweights = TRUE, header = TRUE, mlab=mlabfun("Random Effect Model, IV", model_combined),
       psize=1, rows =  1:length(es_combined$yi), alim = c(log(0.3), log(7)),
       shade = "zebra", xlab = "Odds Ratio (log scale)",
       ilab = paste(es_combined$events, " /", es_combined$n, sep = "")
       , ilab.xpos = -1.5, xlim=c(-4, 3.5), ylim=c(-3, model_combined$k+3), cex = 1.1,
       col = "black", lwd = 2)

# Move the text slightly to the right
text(-1.5, model_combined$k + 1.7, "Events/Total", cex = 1.1, font = 2)
text(-1.5, -2, paste(total_events, " /", total_n, sep = ""), cex = 1.1, font = 2)
```

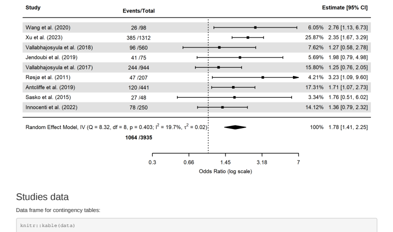

Studies data

Data frame for contingency tables:

```
knitr::kable(data)
```

study	high_tn_diseased	high_tn_survived	low_tn_diseased	low_tn_survived
Innocenti et al. (2022)	39	73	39	99
Sasko et al. (2015)	20	13	7	8
Antcliffe et al. (2019)	89	201	31	120
Røsjø et al. (2011)	43	123	4	37
Vallabhajosyula et al. (2017)	222	623	22	77
Jendoubi et al. (2019)	25	15	16	19
Vallabhajosyula et al. (2018)	88	416	8	48
Xu et al. (2023)	338	699	47	228

Data frame for Contrast-based tables:

```
knitr::kable(data_contrast)
```

study	OR	p	survivor_n	diseased_n	n
Wang et al. (2020)	2.756	0.026	72	26	98

Risk of Bias data:

```
RoB <- read_xlsx("RoB.xlsx", sheet = 1)
trafficlight_rob <- rob_traffic_light(data = RoB,
                                      tool = "QUIPS", overall = FALSE,
                                      psize = 10)

rob_save(trafficlight_rob, "rob_fig_unadj.png")

print(trafficlight_rob)
```

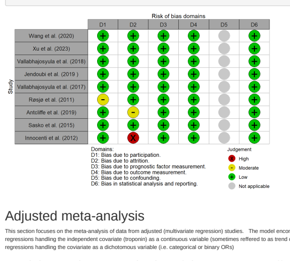

Adjusted meta-analysis

This section focuses on the meta-analysis of data from adjusted (multivariate regression) studies. The model encompasses effect measures from regressions handling the independent covariate (troponin) as a continuous variable (sometimes reffered to as trend or dose-response ORs), and regressions handling the coviariate as a dichotomous variable (i.e. categorical or binary ORs)

Load data and Homogenization of dose-response effect measures

Since the studies report ORs on different scales for the continuous troponin variable, we need to standardize them to a common objective scale.

One challenge arises with the study by Masson et al. (2016), which reports ORs per standard deviation (SD). As the SD is not directly provided, we must approximate it using information from the text to calculate the OR per 1-unit increase.

```
#Load data from adjusted (multivariate) estimates
multivariate <- read_excel("multivariate.xlsx", sheet = 1)
multivariate2 <- read_excel("multivariate.xlsx", sheet = 2)

#Filter the Masson et al. subgroups
masson_groups <- multivariate %>%
  filter(study %in% c("Masson et al. (2016)[No shock group]", "Masson et al. (2016)[Shock group]"))

#Use a fixed effect model to impute an effect measure for the entire cohort
es_masson <- conv.wald(out= OR, pval = p, n = n, data=masson_groups, transf=log)
masson_FEM <- rma(yi, vi, data = es_masson, method = "FE")
masson_OR <- exp(masson_FEM$beta)
masson_p <- masson_FEM$pval

#Include a new row with the pooled estimate for the entire cohort
masson_summarized <- multivariate %>%
  filter(study %in% c("Masson et al. (2016)[No shock group]", "Masson et al. (2016)[Shock group]")) %>%
  group_by(study) %>%
  summarize(n_sum = sum(n), events_sum = sum(events))

new_row_values <- list(
  study = "Masson et al. (2016)",
  OR = masson_OR,
  `Units from ng/L` = "10^0",
  p = masson_p,
  n = sum(masson_summarized$n_sum),
  events = sum(masson_summarized$events_sum)
)

#Add the row to the table
multivariate <- multivariate %>%
  add_row(.data = ., !!!new_row_values)

#Filter the dataframe
multivariate <- multivariate %>%
  filter(!(study %in% c("Masson et al. (2016)[No shock group]", "Masson et al. (2016)[Shock group]")))

##Once we have computed a common OR per 1 SD increase for the cohort, we proceed to escalate this effect measure to an OR per 1 unit increase

#Define functions to calculate an arithmetic standard deviation from Q1, Q2 and Q3.
#Mathematical concepts about this functions are accessible in the next Segment of the supplement)
calculate_mean <- function(Q1, median, Q3) {
  mean_approx <- (Q1 + median + Q3) / 3
  mn <- mean_approx
  return(mn)
}

calculate_standard_deviation <- function(n, Q1, Q3) {
  sd_percentile <- 0.75 * n - 0.125
  phi_inverse <- qnorm(sd_percentile / (n + 0.25))
  sd <- (Q3 - Q1) / (2 * phi_inverse)
  return(sd)
}
calculate_sd_pooled <- function(n_values, s_values) {
  df_values <- n_values - 1
  numerator <- sqrt(
    sum(
      df_values * s_values^2
    )
  )
  denominator <- sqrt(sum(n_values) - length(n_values))
  sd_pooled <- numerator / denominator
  return(sd_pooled)
}

#Load data on quantiles as reported (by arms)
masson_data <- read_excel("masson.xlsx")
masson_data <- masson_data %>%
  mutate(
    SD = calculate_standard_deviation(n, Q1, Q3),
    m = calculate_mean(Q1, median, Q3)
  )

# Filter rows for albumin and crystalloid groups separately
no_shock_data <- masson_data %>%
  filter(group %in% c("no_shock_albumin", "no_shock_crystalloid"))
shock_data <- masson_data %>%
  filter(group %in% c("shock_albumin", "shock_crystalloid"))

#Use a fixed effect model to impute a common mean for the entire arm
no_shock_FE <- rma(measure = "MN", mi = m, sdi = SD, ni = n, data = no_shock_data, method = "FE")
shock_FE <- rma(measure = "MN", mi = m, sdi = SD, ni = n, data = shock_data, method = "FE")
no_shock_m <- coef(no_shock_FE)
shock_m <- coef(shock_FE)

#Impute a common SD for the entire arm
no_shock_SD <- calculate_sd_pooled(no_shock_data$n, no_shock_data$SD)
shock_SD <- calculate_sd_pooled(shock_data$n, shock_data$SD)
SD_by_arm <- data.frame(
  group = c("shock", "no_shock"),
  SD = c(shock_SD, no_shock_SD),
  n = c(sum(shock_data$n), sum(no_shock_data$n))
)

#Impute a common SD for the entire cohort
masson_SD <- calculate_sd_pooled(SD_by_arm$n, SD_by_arm$SD)

#re-scale OR per 1 SD increase, to OR per 1 unit increase
multivariate$OR[multivariate$study == "Masson et al. (2016)"] <- masson_OR ^ (1/no_shock_SD)
```

After deriving the effect measures for all cohorts, we proceed to rescale the dose-response ORs (OR per 1-unit troponin increase) to a clinically significant scale of 50 ng/L, as indicated in the text.

```
#Read the notation of the dataframe into R notation
multivariate <- multivariate %>%
  mutate(`Units from ng/L` = gsub("\\^", "e", `Units from ng/L`))
multivariate <-  multivariate %>%
  mutate(
    `Units from ng/L` = as.numeric(multivariate$`Units from ng/L`)/10
  )

#Raise OR to convert them into ng/L
multivariate <- multivariate %>%
  mutate(OR_ngL = OR^(`Units from ng/L`))

#Now compute the OR per 50 ng/L
multivariate <- multivariate %>%
  mutate(OR_50ngL = OR_ngL^(50))
knitr::kable(multivariate)
```

study	OR	Units from ng/L	p	n	events	OR_ngL	OR_50ngL
Kim et al. (2020)	0.995000	0.001	0.7400000	778	233	0.999995	9.997494e-01
Vallabhajosyula et al. (2017)	1.400000	1.000	0.0400000	944	244	1.400000	2.024892e+07
Masson et al. (2016)	1.002203	1.000	0.1292471	955	253	1.002203	1.116327e+00

```
#Standardize the column names in order to bind both dataframes
multivariate2 <- multivariate2 %>%
  mutate(OR_50ngL = OR)
```

Since “Vallabhajosyula et al. (2017)” log-transformed the troponin variable, the respective OR represents and increment in the logit per 10-fold increase in troponin (ng/mL) . Once again we will estimate the OR of a dependent variable increase of 50ng/L from a baseline of 10 ng/L, since this is representative of the differences in the medians of the groups normal troponin vs elevated troponin (see the *Protocol*). The mathematical proof of this transformation is provided in **(2)** The Maximum Likelihood estimator for the OR is given by:

$$OR\{f(x+c), f(x)\} = \exp\left[\hat{B}_i \cdot (f_i(x_i + c_i) - f_i(x_i))\right]$$

and the The Maximum Likelihood estimator for its confidence interval is given by:

$$\exp\left[\hat{B}_i \cdot (f_i(x_i + c_i) - f_i(x_i)) \pm z_{1-\alpha/2}(f_i(x_i + c_i) - f_i(x_i))\hat{SE}(\hat{B}_i)\right]$$

```
#Calculate OR, given that the reported OR is 1.4, the p value 0.04, and the f(x) function is log10:
b1 = log(1.4)
x = 0.01
c = 0.05
b = (x+c)/(x)
OR_Vallabhajosyula = exp(b1 * ( log10(x+c) - log10(x) ) )

#Replace the new OR in the dataframe
multivariate$OR_50ngL[multivariate$study == "Vallabhajosyula et al. (2017)"] <- OR_Vallabhajosyula
```

Model creation

Having all effect measures on the same scale of troponin variation (OR per 50ng/L), we can use a Random effect model to pool the dose-response ORs with the binary OR.

```
#Bind the dataframes
multivariate <- bind_rows(multivariate, multivariate2)

#Log-transform the point estimate and variance for meta-analytic purposes
es_multivariate <- conv.wald(out= OR_50ngL, pval = p, n = n, data=multivariate, transf=log)

#Define objects for the plot
total_events <- sum (es_multivariate$events)
total_n <- sum (es_multivariate$n)

# Create a random-effects model
model <- rma(yi, vi, data = es_multivariate, slab = es_multivariate$study)

# Create the forest plot with various customization
forest(model, slab = es_multivariate$study, atransf = exp,
       showweights = TRUE, header = TRUE, alim = c(log(0.4), log(5)),
       shade = "zebra", at = seq(-1, 3), xlab = "Odds Ratio (log scale)",
       ilab = paste(es_multivariate$events, "/", es_multivariate$n)
       , ilab.xpos = -1.5, cex=1.1, rows =  1:length(es_multivariate$yi),
       mlab=mlabfun("Random Effect Model, IV", model),
       psize=1, xlim=c(-4, 3.5), ylim=c(-3, model$k+3),
       col = "black", lwd = 2
)

text(-1.5, model$k + 1.7, "(Event/Total)", cex = 1.1, font = 2)
text(-1.5, -2, paste(total_events, "/", total_n), cex = 1.1, font = 2)
```

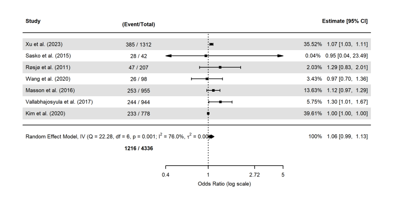

Sensitivity analysis

We run a sensitivity analysis only consideringbinary-troponin adjusted ORs:

```
multivariate2 <- read_xlsx("multivariate.xlsx", sheet = 2)
es_multivariate2 <- conv.wald(out= OR, pval = p, n = n, data=multivariate2, transf=log)
model <- rma(yi, vi, data = es_multivariate2, slab = es_multivariate2$study)
total_events <- sum(es_multivariate2$events)
total_n <- sum(es_multivariate2$n)

# Create the forest plot with various customization
forest(model, slab = es_multivariate2$study, atransf = exp,
       showweights = TRUE, header = TRUE,
       shade = "zebra", at = seq(-1, 3), xlab = "Odds Ratio (log scale)",
       ilab = paste(es_multivariate2$events, "/", es_multivariate2$n)
       , ilab.xpos = -1.5,  cex=1, rows =  1:length(es_multivariate2$yi),
       mlab=mlabfun("Random Effect Model, IV", model),
       psize=1, xlim=c(-4, 3.5), ylim=c(-3, model$k+3)
)

text(-1.5, model$k + 1.7, "(Event/Total)", cex = 1, font = 2)
text(-1.5, -2, paste(total_events, "/", total_n), cex = 1, font = 2)
```

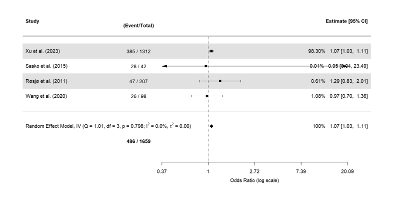

Studies data

The dataframes extracted from the studies is presented here.

```
multivariate <- read_xlsx("multivariate.xlsx", sheet = 1)
knitr::kable(multivariate)
```

study	OR	Units from ng/L	p	n	events
Kim et al. (2020)	0.995	10^-3	0.74	778	233
Masson et al. (2016)[No shock group]	1.100	10^0	0.51	417	94
Masson et al. (2016)[Shock group]	1.200	10^0	0.15	538	159
Vallabhajosyula et al. (2017)	1.400	10^0	0.04	944	244

```
multivariate2 <- read_xlsx("multivariate.xlsx", sheet = 2)
knitr::kable(multivariate2)
```

study	OR	p	n	events
Wang et al. (2020)	0.971	0.86300	98	26
Røsjø et al. (2011)	1.290	0.26000	207	47
Sasko et al. (2015)	0.950	0.97500	42	28
Xu et al. (2023)	1.070	0.00015	1312	385

Risk of Bias data:

```
RoB <- read_xlsx("RoB.xlsx", sheet = 2)
trafficlight_rob <- rob_traffic_light(data = RoB,
                                      tool = "QUIPS",
                                      psize = 10)

rob_save(trafficlight_rob, "rob_fig_adj.png")

print(trafficlight_rob)
```

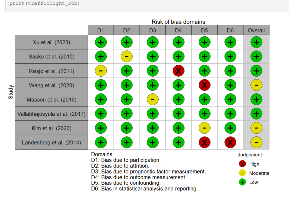

Mean difference data meta-analysis

For the quantitative analysis of studies reporting distribution differences in serum troponin levels for the survivor vs non-survivor group, we will run a Standardized Mean Difference (SDM) model.
This requires first the collection of arithmetic means and standard deviation for all studies.

Load data

```
continuous1 <- read_xlsx("continuous.xlsx", sheet = "Sheet 1")
continuous2 <- read_xlsx("continuous.xlsx", sheet = "sheet 2")
```

Calculation of Arithmetic statistics

Where aggregate data was reported in the form Q2(Q3-Q1), arithmetic mean and standard deviation of the skewed data was calculated with the following. **(3)**

$$\bar{x} \approx \frac{q_1 + q_2 + q_3}{3}$$

$$SD \approx \frac{q_3 - q_1}{2\Phi^{-1}\left(\frac{0.75n - 0.125}{n + 0.25}\right)}$$

where
* bar_x: media of raw (skewed) distribution
* SD: standard deviation of raw (skewed) distribution
* n: sample size

```
#Define the functions for calculating arithmetic statistics
calculate_mean <- function(Q1, median, Q3) {
  mean_approx <- (Q1 + median + Q3) / 3
  mn <- mean_approx
  return(mn)
}

calculate_standard_deviation <- function(n, Q1, Q3) {
  sd_percentile <- 0.75 * n - 0.125
  phi_inverse <- qnorm(sd_percentile / (n + 0.25))
  sd <- (Q3 - Q1) / (2 * phi_inverse)
  return(sd)
}

#Apply to the dataframe
continuous2 <- continuous2 %>%
  mutate(
    survivor_m = calculate_mean(survivor_Q1, survivor_median, survivor_Q3),
    survivor_SD = calculate_standard_deviation(survivor_n, survivor_Q1, survivor_Q3),
    diseased_m = calculate_mean(diseased_Q1, diseased_median, diseased_Q3),
    diseased_SD = calculate_standard_deviation(diseased_n, diseased_Q1, diseased_Q3)
  )

# merge the dataframes by their arithmetic statistics
continuous_data <- bind_rows(continuous1, continuous2)
```

SMD model

```
#Calculate the SMD metrics
es_continuous <- escalc(measure = "SMD",
                        m1i = diseased_m, sd1i = diseased_SD,
                        m2i = survivor_m, sd2i = survivor_SD,
                        n1i = diseased_n, n2i = survivor_n,
                        data = continuous_data)


#Define the Random Effect Model
continuous_model <- rma(yi, vi, data = es_continuous, method = "REML", slab = es_continuous$study)


# Display forest plot
forest(
  continuous_model, xlim=c(-5, 3.5), ylim=c(-3, continuous_model$k+3), order=Trop,
  slab = es_continuous$study,
  showweights = TRUE,
  header = TRUE,
  shade = "zebra",
  at = seq(-1, 2),
  ilab = paste(es_continuous$diseased_n, "/", es_continuous$survivor_n + es_continuous$diseased_n),
  ilab.xpos = -1.5,
  cex=1.1, rows =  1:length(es_continuous$yi),
  mlab=mlabfun("Random Effect Model, IV", continuous_model),
  psize=1, col = "black", lwd = 2
)

#create Total sample size
total_n <- sum(es_continuous$diseased_n + es_continuous$survivor_n)
total_events <- sum(es_continuous$diseased_n)

# Move the text slightly to the right
text(-1.5, continuous_model$k + 1.7, "(Event/Total)", cex = 1.1, font = 2)
text(-1.5, -2, paste(total_events, "/", total_n), cex = 1.1, font = 2)
```

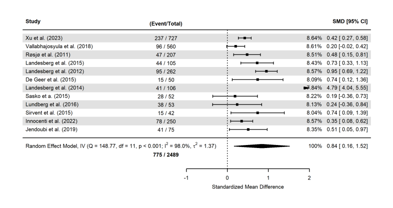

Interpret the model

In order to interpret the SMD model we will cancel out the pooled standard deviation from the pooled estimate as exemplified by Fu et al. **(4)**. We will use Xu et al. cohort´s standard deviation as a metric representative of the SD for the corpus of studies, since it is the largest of the studies, and corresponds to a Troponin T essays -which constitute most of the model´s sample.

Where the pooled SD is given by:

$$\{sdpi\} = \sqrt{\frac{(n_1 - 1)\cdot\{sd1\}^2 + (n_2 - 1)\cdot\{sd2\}^2}{n_1 + n_2 - 2}}$$

Where:

n_1, n_2 are the sample sizes of groups 1 and 2, respectively,
$\{sd1\}, \{sd2\}$ are the standard deviations of groups 1 and 2, respectively.

```
# Subset the data for the study "Xu et al. (2023)"
Xu_data <- subset(continuous_data, study == "Xu et al. (2023)")
diseased_SD <- Xu_data$diseased_SD
survivor_SD <- Xu_data$survivor_SD
diseased_n <- Xu_data$diseased_n
survivor_n <- Xu_data$survivor_n

#Define function to calculate a common SD
calculate_sd_pooled <- function(sd1, sd2, n1, n2) {
  sdpi <- sqrt(((n1 - 1) * sd1^2 + (n2 - 1) * sd2^2) / (n1 + n2 - 2))
  return(sdpi)
}

# Calculate sdpi using the calculate_sdpi function
Xu_SD <- calculate_sd_pooled(diseased_SD, survivor_SD, diseased_n, survivor_n)

Xu_SD
```

```
## [1] 125.8179
```

Multiplying the model estimate by the pooled SD computes an estimation for the mean difference of troponin distributions.

```
#Extract point estimate (CI)
MD <- Xu_SD * coef(continuous_model)
MD_low <- Xu_SD * continuous_model$ci.lb
MD_up <- Xu_SD * continuous_model$ci.ub
cat("Mean Difference (MD): ", MD, "\nMD Lower Bound: ", MD_low, "\nMD Upper Bound: ", MD_up, "\n")
```

```
## Mean Difference (MD):  105.6141
## MD Lower Bound:  20.4118
## MD Upper Bound:  190.8163
```

Heterogeneity investigation

If we regard “Landesberg et al. (2014)” as an outlier:

```
#Calculate the SMD metrics
es_continuous <- escalc(measure = "SMD",
                        m1i = diseased_m, sd1i = diseased_SD,
                        m2i = survivor_m, sd2i = survivor_SD,
                        n1i = diseased_n, n2i = survivor_n,
                        data = continuous_data)

#Remove the outlier
es_continuous <-  es_continuous %>% filter(study != "Landesberg et al. (2014)")

#Define the Random Effect Model
continuous_model <- rma(yi, vi, data = es_continuous, method = "REML",  slab = es_continuous$study)


#Define the Random Effect Model
continuous_model <- rma(yi, vi, data = es_continuous, method = "REML", slab = es_continuous$study)


# Display forest plot
forest(
  continuous_model, xlim=c(-5, 3.5), ylim=c(-3, continuous_model$k+3), order=Trop,
  slab = es_continuous$study,
  showweights = TRUE,
  header = TRUE,
  shade = "zebra",
  at = seq(-1, 2),
  ilab = paste(es_continuous$diseased_n, "/", es_continuous$survivor_n + es_continuous$diseased_n),
  ilab.xpos = -1.5,
  cex=1, rows =  1:length(es_continuous$yi),
  mlab=mlabfun("Random Effect Model, IV", continuous_model),
  psize=1,
)

#create Total sample size
total_n <- sum(es_continuous$diseased_n + es_continuous$survivor_n)
total_events <- sum(es_continuous$diseased_n)

# Move the text slightly to the right
text(-1.5, continuous_model$k + 1.7, "(Event/Total)", cex = 1, font = 2)
text(-1.5, -2, paste(total_events, "/", total_n), cex = 1, font = 2)
```

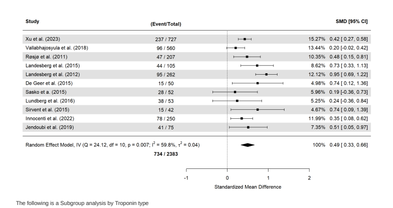

The following is a Subgroup analysis by Troponin type

```
es_continuous <- escalc(measure = "SMD",
                        m1i = diseased_m, sd1i = diseased_SD,
                        m2i = survivor_m, sd2i = survivor_SD,
                        n1i = diseased_n, n2i = survivor_n,
                        data = continuous_data)
continuous_model <- rma(yi, vi, data = es_continuous, method = "REML", slab = es_continuous$study)

# Display forest plot
forest(
  continuous_model, xlim=c(-5, 3.5), ylim=c(-3, 22), order=Trop,
  slab = es_continuous$study,
  showweights = TRUE,
  header = TRUE,
  shade = "zebra",
  at = seq(-1, 2),
  ilab = paste(es_continuous$diseased_n, "/", es_continuous$survivor_n + es_continuous$diseased_n),
  ilab.xpos = -1.5,
  cex=1,
  mlab=mlabfun("REM for All Studies (IV)", continuous_model),
  psize=1,
  rows=c(3:4,9:18)
)

### set font expansion factor (as in forest() above) and use a bold font
op <- par(cex=1, font=2)

#create Total sample size
total_n <- sum(es_continuous$diseased_n + es_continuous$survivor_n)
total_events <- sum(es_continuous$diseased_n)

# Add heading
text(-1.5, 20.8, "(Event/Total)", cex = 1, font = 2)


### add text for the subgroups
text(-5, c(5.5,19.2), pos=4, c("cTnI", "cTnT"))

### set par back to the original settings
par(op)

### fit random-effects model in the three subgroups
res.yes <- rma(yi, vi, subset=(Trop=="cTnT"), data=es_continuous)
res.no <- rma(yi, vi, subset=(Trop=="cTnI"),     data=es_continuous)

addpoly(res.yes, row=7.5, mlab=mlabfun("RE Model for Subgroup", res.yes))
addpoly(res.no, row= 1.5, mlab=mlabfun("RE Model for Subgroup", res.no))

### fit meta-regression model to test for subgroup differences
res <- rma(yi, vi, mods = ~ Trop, data=es_continuous)

### add text for the test of subgroup differences
text(-5, -2.2, pos=4, cex=1, bquote(paste("Test for Subgroup Differences: ",
                                          Q[M], " = ", .(fmtx(res$QM, digits=2)),
                                          ", df = ", .(res$p - 1), ", ",
                                          .(fmtp(res$QMp, digits=2, pname="p",
                                                 add0=TRUE, sep=TRUE, equal=TRUE)))))
```

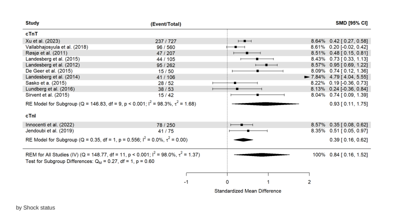

by Shock status

```
es_continuous <- escalc(measure = "SMD",
                        m1i = diseased_m, sd1i = diseased_SD,
                        m2i = survivor_m, sd2i = survivor_SD,
                        n1i = diseased_n, n2i = survivor_n,
                        data = continuous_data)
continuous_model <- rma(yi, vi, data = es_continuous, method = "REML", slab = es_continuous$study)

# Display forest plot
forest(
  continuous_model, xlim=c(-5, 3.5), ylim=c(-3, 22), order=shock,
  slab = es_continuous$study,
  showweights = TRUE,
  header = TRUE,
  shade = "zebra",
  at = seq(-1, 2),
  ilab = paste(es_continuous$diseased_n, "/", es_continuous$survivor_n + es_continuous$diseased_n),
  ilab.xpos = -1.5,
  cex=1,
  mlab=mlabfun("REM for All Studies (IV)", continuous_model),
  psize=1,
  rows=c(3:6,11:18)
)

### set font expansion factor (as in forest() above) and use a bold font
op <- par(cex=1, font=2)

#create Total sample size
total_n <- sum(es_continuous$diseased_n + es_continuous$survivor_n)
total_events <- sum(es_continuous$diseased_n)

# Add heading
text(-1.5, 20.8, "(Event/Total)", cex = 1, font = 2)


### add text for the subgroups
text(-5, c(7.5,19.2), pos=4, c(
  "Not shock-only", "Shock-only"))




### fit random-effects model in the three subgroups
res.yes <- rma(yi, vi, subset=(shock=="yes"), data=es_continuous)
res.no <- rma(yi, vi, subset=(shock=="no"),     data=es_continuous)

addpoly(res.yes, row=9, mlab=mlabfun("RE Model for Subgroup", res.yes))
addpoly(res.no, row= 1.5, mlab=mlabfun("RE Model for Subgroup", res.no))

### fit meta-regression model to test for subgroup differences
res <- rma(yi, vi, mods = ~ shock, data=es_continuous)

### add text for the test of subgroup differences
text(-5, -2.2, pos=4, cex=1, bquote(paste("Test for Subgroup Differences: ",
                                          Q[M], " = ", .(fmtx(res$QM, digits=2)),
                                          ", df = ", .(res$p - 1), ", ",
                                          .(fmtp(res$QMp, digits=2, pname="p",
                                                 add0=TRUE, sep=TRUE, equal=TRUE)))))
```

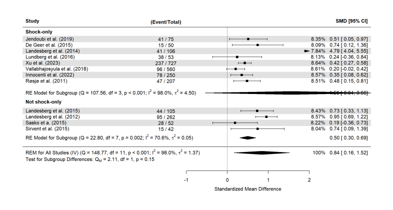

Studies data

The dataframes extracted from the studies are presented here:

```
continuous1 <- read_xlsx("continuous.xlsx", sheet = "Sheet 1")
knitr::kable(continuous1)
```

study	diseased_m	diseased_SD	diseased_n	survivor_m	survivor_SD	survivor_n	shock	Trop
Sirvent et al. (2015)	1156	1804	15	325	363	27	no	cTnT
Lundberg et al. (2016)	143	444	38	51	85	15	yes	cTnT
Sasko et a. (2015)	4500	11300	28	2500	9500	24	no	cTnT
Landesberg et al. (2014)	410	81	41	59	67	65	yes	cTnT

```
continuous2 <- read_xlsx("continuous.xlsx", sheet = "sheet 2")
knitr::kable(continuous2)
```

study	diseased_median	diseased_Q1	diseased_Q3	diseased_n	survivor_median	survivor_Q1	survivor_Q3	survivor_n	shock	Trop
De Geer et al. (2015)	121	39	530.0	15	40	24	119	35	yes	cTnT
Landesberg et al. (2012)	150	60	250.0	95	40	10	110	167	no	cTnT
Landesberg et al. (2015)	140	50	230.0	44	50	8	120	61	no	cTnT
Røsjø et al. (2011)	54	22	227.0	47	36	15	111	160	no	cTnT
Jendoubi et al. (2019)	57	12	115.0	41	23	10	54	34	yes	cTnI
Innocenti et al. (2022)	110	20	452.5	78	70	20	250	172	no	cTnI
Vallabhajosyula et al. (2018)	50	20	132.5	96	50	30	90	464	no	cTnT
Xu et al. (2023)	80	30	280.0	237	60	30	140	490	no	cTnT

Risk of Bias data:

```
RoB <- read_xlsx("RoB.xlsx", sheet = 3)
trafficlight_rob <- rob_traffic_light(data = RoB,
                                      tool = "QUIPS",
                                      psize = 10)

rob_save(trafficlight_rob, "rob_fig_SMD.png")

print(trafficlight_rob)
```

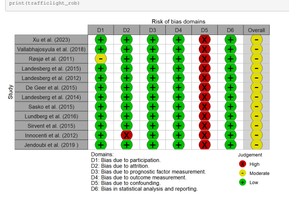

Publication Bias

Funnel plots to asses publication bias.

```
#funnel unadjusted (univariate) model
funnel(
  model_combined,
  level = c(90, 95, 99),
  shade = c("white", "gray55", "gray75"),
  digits = 3L,
  ylim = c(0, .8),
  atransf = exp,
  at = log(c(.125, .25, .5, 1, 2, 4, 8)),
  legend = TRUE,
  xlab = "Odd Ratio",
  ylab = "Standard Error",
   label= "out",
)
```

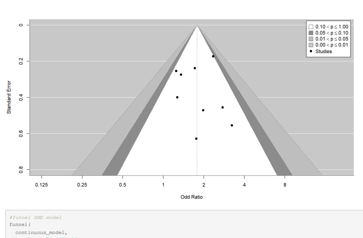


```
#funnel SMD model
funnel(
  continuous_model,
  level = c(90, 95, 99),
  shade = c("white", "gray55", "gray75"),
  digits = 3L,
  ylim = c(0, .8),
  atransf = exp,
  at = log(c(.125, .25, .5, 1, 2, 4, 8)),
  legend = FALSE,
  xlab = "SMD",
  ylab = "Standard Error",
   label= "out",
)
```

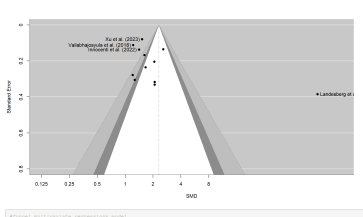


```
#funnel multivariate regressions model
funnel(
  model,
  level = c(90, 95, 99),
  shade = c("white", "gray55", "gray75"),
  digits = 3L,
  ylim = c(0, .8),
  atransf = exp,
  at = log(c(.125, .25, .5, 1, 2, 4, 8)),
  legend = FALSE,
  xlab = "Odd Ratio",
  ylab = "Standard Error",
   label= "out",
)
```

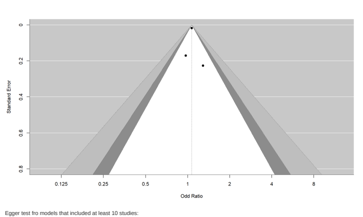

Egger test fro models that included at least 10 studies:

```
regtest(continuous_model)
```

```
##
## Regression Test for Funnel Plot Asymmetry
##
## Model:     mixed-effects meta-regression model
## Predictor: standard error
##
## Test for Funnel Plot Asymmetry: z =  1.9280, p = 0.0539
## Limit Estimate (as sei -> 0):   b = -0.5569 (CI: -2.0956, 0.9817)
```

Risk of Bias assesment algorithm

For the RoB assessment we used 6 domains, 3 of which were considered key domains. The classification for each domain in low, moderate or high RoB, was decided as follows (based on the Quality In Prognosis Studies tool):

D1. Study participation (key domain): based on the relationship between troponins and outcome, according to the description of the sample and population of interest. High: very like to be different for participants and eligible non participants. Moderate: may be different for participants and eligible non participants. Low: very like to be different for participants and eligible non participants.

D2. Study attrition: based on the relationship between troponins and outcome, according to the description of follow-up of the participants. High: very like to be different for for completing and noncompleting participants. Moderate: may be different for for completing and noncompleting participants. Low: unlikely to be different for for completing and noncompleting participants

D3. Prognostic Factor Measurement (key domain): based on the method used for the measurement of troponins, and whether it is the same for all the participants. High:The measurement is very likely to be different for different levels of the outcome of interest. Moderate: The measurement may be different for different levels of the outcome of interest. Low: The measurement is unlikely to be different for different levels of the outcome of interest

D4 Outcome Measurement: based on whether the definition and measurement of outcome (mortality in “X” time) was the same for all participants. High: The measurement of the outcome is very likely to be different related to the baseline level of troponins. Moderate: The measurement of the outcome may be different related to the baseline level of troponins. Low: The measurement of the outcome is unlikely to be different related to the baseline level of troponins.

D5 Study Confounding (key domain): for this domain we defined 3 core adjustment factors:

a. Severity (eg shock or severity score -APACHE, SOFA, etc)
b. Age
c. Cardiac and renal comorbidities

High: only 1 or none of these factors was successfully adjusted. Moderate 2 of these factors were successfully adjusted. Low: all 3 of them were successfully adjusted.

D6. Statistical Analysis and Reporting (key domain): based on the quality of the statistical analysis and whether all primary outcomes were reported. Selective reporting of results was defined when a regression analysis only reports OR for some of its variables and not all of them. High: The reported results are very likely to be spurious or biased related to analysis or reporting. Moderate: The reported results may be spurious or biased related to analysis or reporting. Low: The reported results are unlikely to be spurious or biased related to analysis or reporting.

Finally, **the overall RoB** was assigned based on the 3 key domains: study participation, prognostic factor measurement and study confounding. If all of these were low risk, or 2 low plus 1 moderate risk, the overall assessment was low RoB. If there was high RoB for 1 domain, or moderate RoB for at least 2 domains (with the rest as low RoB), overall moderate RoB was assigned. For at least 2 domains with high RoB, we classified the study as high RoB.

References

1. Hu D, Wang C, O’Connor AM (2020) A method of back-calculating the log odds ratio and standard error of the log odds ratio from the reported group-level risk of disease. PLoS ONE 15 (3): e0222690. https://doi.org/10.1371/journal. pone.0222690
2. Elswick RK Jr, Schwartz PF, Welsh JA. Interpretation of the odds ratio from logistic regression after a transformation of the covariate vector. Stat Med. 1997 Aug 15;16(15):1695-703. doi: 10.1002/(sici)1097-0258(19970815)16:15<1695::aid-sim601>3.0.co;2-v. PMID: 9265694.
3. Wan, X., Wang, W., Liu, J. et al. Estimating the sample mean and standard deviation from the sample size, median, range and/or interquartile range. BMC Med Res Methodol 14, 135 (2014). https://doi.org/10.1186/1471-2288-14-135
4. Fu R, Vandermeer BW, Shamliyan TA, O’Neil ME, Yazdi F, Fox SH, Morton SC. Handling Continuous Outcomes in Quantitative Synthesis. 2013 Jul 25. In: Methods Guide for Effectiveness and Comparative Effectiveness Reviews [Internet]. Rockville (MD): Agency for Healthcare Research and Quality (US); 2008–. PMID: 24006546.
